# Genomic medicine in Africa: promise, problems and prospects

**DOI:** 10.1186/gm528

**Published:** 2014-02-24

**Authors:** Ambroise Wonkam, Bongani M Mayosi

**Affiliations:** 1 Division of Human Genetics, Department of Clinical Laboratory Sciences, National Health Laboratory Service and University of Cape Town, Anzio Road, Observatory – 7925, Cape Town, South Africa; 2 Department of Medicine, University of Cape Town and Groote Schuur Hospital, Anzio Road, Observatory – 7925, Cape Town, South Africa

## The promise

Remarkable progress has been made in using genomic information to determine how genes are regulated, and how they interact with each other and with the environment to control complex biochemical functions of living organisms in health and disease [1]. This information will have major benefits for the prevention, diagnosis and management of many diseases, including communicable and genetic diseases. In Africa, where infectious diseases are highly prevalent, research on pathogen genomes has enhanced our understanding of disease transmission, virulence mechanisms and avoidance of host defenses [2]. It is anticipated that this information will enable the development of new diagnostic tests, vaccines and therapeutic agents; it is also likely to lead to new approaches for vector control, and reveal why individuals and populations vary in their susceptibility to infectious diseases [1].

This commentary focuses on problems and prospects for the use of new genomic knowledge in improving the health of Africans. The prospects for genomic medicine in Africa have been enhanced by major initiatives that are led by international funding agencies and academics, such as the Malaria Genomic Epidemiology Network (MalariaGEN) (http://www.malariagen.net/) and the Human Heredity and Health in Africa (H3Africa) program (http://h3africa.org/h3africa_whitepaper.pdf). These multi-national consortia have started to build the capacity in research skills and overcome the barriers for the use of genomics to address the disease burden of Africans. The time has come for African governments to heed the call of the World Health Organization to embrace genomics for the benefit of their populations.

## The problems

There is a danger that new developments in genomics will increase the disparity in healthcare within countries, and between developed and developing countries, due to the high cost of implementing research findings in the clinic [1]. Inequalities in healthcare may be further exacerbated by the current intellectual property regime unless care is taken to ensure that knowledge produced through genomics research becomes a public good [3]. Furthermore, the limited biotechnology and information technology infrastructure in many African countries hinders the participation of researchers and clinicians in genomic medicine. There is a lack of market incentives for the private sector to invest in genomics research directed towards neglected diseases of the world’s poorest people. Therefore, the potential for genomics research to combat these diseases will only be realized through an enforceable global mechanism for public investment in health research on such diseases [3]. Indeed, more than one billion people in developing countries suffer from one or more neglected infectious diseases, most of which are found in Africa. Battling neglected infectious diseases is an important priority of the Bill & Melinda Gates Foundation (http://www.gatesfoundation.org), which has committed more than US$1.02 billion for research on methods of treatment and prevention, using genomic technologies in some cases.

Finally, the high cost of genomics and lack of technological capacity is compounded by a relatively low level of public and professional understanding of genomics in African countries [4]. There is, therefore, a need for African societies to prepare for the genomics era and its consequences through education at all levels, with particular emphasis on improving science education and the introduction of the principles of biomedical ethics to school children [1]. H3Africa draws increased attention to a number of longstanding and emerging issues in genetic and genomic research, such as informed consent, community engagement, privacy and confidentiality, use of genetic information, governance of biorepositories, reciprocity/benefit sharing, and return of research results and incidental findings. These issues are highlighted by genome-wide association studies or whole exome/genome sequencing that will be carried out in many projects. Unfortunately, there is a dearth of published research on the views of Africans and African communities concerning participation in genomic research, and virtually no information on their perspectives regarding several of these issues [4].

## The prospects

Simple clinical applications of DNA technology could provide immediate benefit for healthcare in many African countries. For example, sickle cell disease (SCD), an inherited condition, imposes a heavy economic burden on health systems in Africa, with nearly two-thirds of the 305,800 infants born annually with SCD worldwide living in Africa [5]. Prenatal genetic diagnosis of SCD would allow the option of termination of pregnancy. In Cameroon, despite the fact that the majority of doctors and parents of SCD children and adult SCD patients agreed with the principle of testing for prenatal genetic diagnosis of SCD (78.7%, 89.8% and 89.2%, respectively), parents (62.5% acceptance) were more in favor of termination of an SCD-affected pregnancy than doctors and adult patients (36.1% and 40.9% acceptance, respectively) [6]. These preliminary data signal potential value-based conflicts, and can inform concrete future policy interventions as genomic medicine is increasingly employed in African countries. Genomic data can also be used in secondary prevention of SCD, and African researchers are participating actively in producing clinically relevant genetic research on SCD. For example, a study from Tanzania has confirmed that polymorphisms in *BCL11A* and *HBS1L-MYB* genes modulate fetal hemoglobin (HbF) levels and thereby modify the severity of SCD [7]. Identifying loci that affect HbF levels could be used to predict an individual’s ability to produce HbF from birth, and prepare a treatment regimen to reduce the severity of SCD. As genomic technology advances, the values and practices of health professionals and community members must be studied in order to ensure culturally appropriate and cost-effective implementation of strategies to improve awareness and early detection, and prevention of complications of SCD [6].

Although there is sporadic evidence of participation of Africans in researching the genomics of rare monogenic [8] and multifactorial conditions [9], there is little evidence that this improved contribution of African researchers to research has an impact on wider public understanding of, attitudes to, and understanding of genomic medicine. The area where there has been the greatest contribution is in the understanding of the genomic basis of malaria through MalariaGEN, which brings together researchers from 21 countries in Africa and elsewhere [10]. MalariaGEN has made major contributions to overcoming the scientific, ethical and practical challenges to carrying out such studies in Africa where the burden of malaria is greatest. The achievements of MalariaGEN include: analysis of 86,158 exonic single nucleotide polymorphisms in 227 samples, which has allowed the establishment of an open-access web application for the exploration of regional differences in allele frequency and of highly differentiated loci in the *Plasmodium falciparum* genome [10]; reviewing the methodological challenges of genome-wide association studies in Africa, and how these studies will be transformed by new approaches in statistical imputation and large-scale genome sequencing; the exploration of achieving valid consent both from the perspective of protecting individuals and building trust between communities and research groups, and recommendations of ethical data-release policies that provide adequate protection for the research aspirations of developing country scientists (http://www.sanger.ac.uk/research/initiatives/globalhealth/partnerships/malariagen.html).

The prospects for genomic medicine in Africa have been given a major boost by the US National Institutes of Health and the Wellcome Trust, which are investing approximately US$37 million in the H3Africa program over 5 years. The H3Africa consortium has three major goals: to increase the number of African scientists who are internationally competitive in genomics and population-based research; to establish collaborative networks of African investigators pursuing genomics-based disease-oriented projects on conditions that are prevalent in Africa; and to create and expand the infrastructure for genomics research, including bioinformatics and biorepositories on the African continent (http://h3africa.org/h3africa_whitepaper.pdf). This initiative, which was conceived by the African Society of Human Genetics, is set to expand the capacity of African institutions to undertake research in genomic medicine over the next decade.

## A call to action

Improved capabilities of African institutions to undertake research will be realized if ministries of health implement at least three of the key recommendations of the World Health Organization Report on Genomics and World Health [1]. First, capacity building will be achieved most effectively through the development of academic public research and industrial partnerships between developed and developing countries and between developing countries themselves, encouraged by tax or other incentives, and through extensive networking within regions where there is evolving expertise in biotechnology. The H3Africa program has planted the seed for capacity building, which needs to be nurtured by African governments to ensure the long-term sustainability of genomic medicine on the continent. Second, to be able to utilize the vast quantities of genomic data that are being generated, African countries must develop a critical mass of expertise in bioinformatics. There is already a shortage of trained scientists and technicians in this crucially important discipline, even in developed countries. As a first step, countries may decide to examine their current capacity in bioinformatics and identify existing shortfalls. Finally, all African countries need to evolve appropriate national frameworks to consider the ethical implications of genomics research and its applications in their own unique social, cultural, economic and religious context; empirical data from a broad spectrum of stakeholders and the public are needed for the development of effective genomic policies and programs of disease that affect the continent.

## Abbreviations

H3Africa: Human Heredity and Health in Africa; HbF: fetal hemoglobin; MalariaGEN: Malaria Genomic Epidemiology Network; SCD: sickle cell disease.

## Competing interests

The authors declare that they have no competing interests.
